# PD-1/PD-L1 Blockade Therapy for Tumors with Downregulated MHC Class I Expression

**DOI:** 10.3390/ijms18061331

**Published:** 2017-06-21

**Authors:** Michal Šmahel

**Affiliations:** Department of Genetics and Microbiology, Faculty of Science, Charles University, BIOCEV, Průmyslová 595, 25250 Vestec, Czech Republic; smahelm@natur.cuni.cz; Tel.: +420-325-873-921

**Keywords:** PD-1, PD-L1, checkpoint blockade, MHC class I, tumor escape, cancer immunotherapy, biomarker, interferon gamma

## Abstract

The therapy of different advanced-stage malignancies with monoclonal antibodies blocking programmed cell death protein 1 (PD-1)/PD-1 ligand 1 (PD-L1) signaling has had an impressive long-lasting effect in a portion of patients, but in most cases, this therapy was not successful, or a secondary resistance developed. To enhance its efficacy in treated patients, predictive biomarkers are searched for and various combination treatments are intensively investigated. As the downregulation of major histocompatibility complex (MHC) class I molecules is one of the most frequent mechanisms of tumor escape from the host’s immunity, it should be considered in PD-1/PD-L1 checkpoint inhibition. The potential for the use of a PD-1/PD-L1 blockade in the treatment of tumors with aberrant MHC class I expression is discussed, and some strategies of combination therapy are suggested.

## 1. Introduction

The blockade of inhibitory immune checkpoints with monoclonal antibodies contributed to the revival of interest and belief in cancer immunotherapy. After pioneering studies with a cytotoxic T-lymphocyte associated antigen 4 (CTLA-4; cluster of differentiation (CD) 152) blockade that resulted in the Food and Drug Administration (FDA)’s approval of ipilimumab for the treatment of advanced melanoma in 2011, programmed cell death protein 1 (PD-1; CD279)/PD-1 ligand 1 (PD-L1) signaling is in the focus of the current research on, and the development of, anti-tumor therapy in this field. This is because the blocking of PD-1 or PD-L1 molecules exhibited higher efficacy and lower toxicity for several types of human cancers, including melanoma, non-small cell lung cancer (NSCLC), and renal cell cancer (RCC). However, most patients did not respond to the PD-1/PD-L1 blockade, and secondary resistance to this treatment developed in some patients. The mechanisms implicated in this failure are being gradually uncovered, but the biomarkers predicting successful therapy with PD-1/PD-L1 monoclonal antibodies still have not been satisfactorily revealed. The effect of the PD-1 receptor inhibition is usually attributed to the activation of cytotoxic T lymphocytes, and their direct killing of tumor cells producing major histocompatibility complex class I (MHC-I) molecules. Surprisingly, while MHC-I downregulation is one of the most frequent mechanisms of tumor escape from the host’s immune system, little attention has been devoted to surface MHC-I expression in studies of the PD-1/PD-L1 blockade. In this review, we will deal with the relationship between the inhibition of PD-1/PD-L1 signaling and MHC-I expression, and suggest a possible use of the PD-1/PD-L1 blockade for tumors with a reduced MHC-I expression.

## 2. PD-1/PD-L1 Signaling

The PD-1 receptor is an immune checkpoint that limits the activity of immune cells in peripheral tissues, and thus prevents the development of autoimmune reactions. PD-1 was identified in association with programmed cell death in T-cell hybridoma [1], and its function was mainly investigated in T lymphocytes, but it is also expressed in B, natural killer (NK), and NKT cells, as well as dendritic cells (DCs) and macrophages [2]. PD-1 is absent or lowly expressed in resting naïve or memory T cells, but upon the activation of a T-cell receptor (TCR), it is upregulated in about six hours [3]. In tumors, PD-1 is highly expressed in dysfunctional/exhausted effector T cells (both CD8^+^ and CD4^+^) and regulatory T cells (Treg) [4].

PD-1 is a monomeric type I transmembrane protein that belongs to the immunoglobulin (Ig) superfamily, and is composed of an extracellular part containing an IgV-like domain, a transmembrane domain, and a short cytoplasmic tail with an immunoreceptor tyrosine-based inhibitory motif (ITIM) and an immunoreceptor tyrosine-based switch motif (ITSM). PD-1 engagement leads to the phosphorylation of these motifs and the recruitment of the Src homology region 2 domain-containing phosphatase 1 (SHP-1) and SHP-2 [5], which inhibits the TCR/CD28-mediated activation of phosphatidylinositol 3-kinase (PI3K) by the dephosphorylation of CD3 molecules. Thus, PD-1 engagement directly inhibits effector T-cell processes and functions, including proliferation, survival, glucose uptake, cytokine production, and cytotoxicity.

PD-1 binds two ligands from the B7 family: PD-L1 (B7-H1, CD274) [6] and PD-L2 (B7-DC, CD273) [7]. While PD-L1 is widely expressed constitutively both on hematopoietic cells (including macrophages, DCs, and B and T cells) and nonhematopoietic cells (e.g., epithelial and endothelial cells), PD-L2 expression is restricted to immune cells (macrophages, DCs, and mast cells) [8]. PD-L1 is also frequently expressed on tumor cells on various malignancies [9]. While PD-L1 expression is significantly correlated with a poor prognosis in patients with different types of tumors, including renal, gastric, urothelial, ovarian, hepatocellular, pancreatic, and esophageal cancer, there is an inverse correlation in Merkel cell carcinoma and breast cancer. In lung carcinoma, colorectal cancer, and melanoma, both worse and better prognoses were associated with PD-L1 expression. The inconsistency of the prognostic value of PD-L1 could stem from technical issues of the immunohistochemical (IHC) detection of PD-L1, and temporal and spatial factors that can be affected by the heterogeneity of PD-L1 expression in tumors [10].

Two mechanisms for PD-L1 upregulation in malignant cells have been described [11,12]. First, an intrinsic (innate) resistance is caused by constitutive PD-L1 expression in tumor cells that can be induced by oncogenic signaling pathways activated in different tumors, such as PI3K/AKT [13,14], signal transducer and activator of transcription (STAT)-3 [15], epidermal growth factor receptor (EGFR) [16], cyclin-dependent kinase 5 (Cdk5) [17], and MYC pathways [18], or by genetic changes. In lymphomas, an amplification of the PD-L1 gene [19], or its fusion with the MHC class II transactivator CIITA gene, has resulted in PD-L1 overexpression [20]. Furthermore, PD-L1 overexpression has been found in a subtype of gastric cancer associated with the Epstein–Barr virus (EBV) infection, and in cervical cancer associated with the human papillomavirus (HPV) infection, where the enhanced PD-L1 production can also be caused by PD-L1 gene amplification [21,22] or upregulation by EBV-encoded latent membrane protein 1 (LMP1) [23]. Kataoka et al. [24] have identified a novel mechanism of PD-L1 upregulation, characterized by structural variation in the 3′-untranslated region (3′-UTR) of PD-L1 transcripts that is also inducible by the integration of EBV and HPV genomes. Second, in adaptive resistance, PD-L1 expression is induced by specific cytokines, e.g., interferon (IFN)-γ, granulocyte-macrophage colony-stimulating factor (GM-CSF), and interleukin (IL)-4 [25]. These cytokines, in particular IFN-γ, mediate the response of tumor cells to a potentially harmful inflammatory microenvironment.

S. Chikuma [26] has suggested three “immune checkpoints” controlled by the PD-1 receptor that inhibit the reactivity of T lymphocytes: (i) the induction of anergy during the initial activation of T cells; (ii) the inhibition of innate cells (macrophages and DCs) that produce inflammatory cytokines contributing to the differentiation of effector T cells; and (iii) the maintenance of T-cell anergy in peripheral tissues.

## 3. Tumor Escape by MHC-I Downregulation

Tumor cells that present antigenic peptides on MHC-I molecules can be recognized and eliminated by cytotoxic CD8^+^ T lymphocytes (CTL). CTLs are commonly considered major cells acting in cancer immunosurveillance and immunoediting [27]. Tumor cells can escape from the attack of CTLs, especially by the downregulation of tumor antigen(s) (particularly after active immunotherapy), defects in the surface expression of MHC-I molecules, the upregulation of factors conferring a resistance to the cytotoxic effects of immune cells, and/or local immunosuppression. Aberrant MHC-I phenotypes, which have been identified in human tumors, can be characterized by total, haplotype, locus, or allelic loss. Furthermore, in a compound phenotype, these alterations are combined. The genetic changes in genes or chromosomes are responsible for irreversible MHC-I downregulation. If epigenetic modifications or other types of gene expression regulation affect the MHC-I surface expression, the reduction of MHC-I molecules is reversible and can be restored by the induction by different cytokines, especially interferons, or the tumor necrosis factor α (TNF-α) [28].

The major genes whose structural alteration or expression regulation affects surface MHC-I expression are those encoding MHC-I heavy chains, β2-microglobulin, and components of the antigen processing machinery (APM). The APM components include inducible subunits of proteasome (low molecular weight protein (LMP)-2, LMP-7, and LMP-10), the transporters associated with antigen processing (TAP)-1 and TAP-2, which form a heterodimer transporting peptides from the cytosol to the endoplasmic reticulum (ER), and the ER chaperones calnexin, calreticulin, ERp57, and tapasin [29]. These chaperones enable the formation of the trimeric complex, consisting of the MHC-I heavy chain, β2-microglobulin, and a peptide that is then transported through the Golgi apparatus to the cell surface. As all of these proteins are inducible by IFN-γ, defects in the signaling pathways of this cytokine can limit surface MHC-I expression. The proteins involved in aberrant IFN-γ regulation include the transcription factors IFN-regulatory factor 1 (IRF-1) and STAT-1, and the kinases Janus-associated kinase (JAK)-1 and JAK-2 [29].

The downregulation of surface MHC-I expression is a frequent mechanism for immune escape in various human malignancies, ranging from 15% in renal carcinoma to 93% in lung cancer. In most types of tumors derived from epithelia, MHC-I molecules are reduced in more than 75% of patients [28]. The majority of MHC-I aberrations are reversible, and are associated with APM defects. Genetic alterations in MHC-I heavy chains or the light chain (β2-microglobulin) have been found in about one third of human tumors [30]. In ovarian cancer, it has been hypothesized that the inhibition of antigen presentation by the reduction of MHC-I molecules and the suppression of T cells by PD-L1 expression are two mutually exclusive mechanisms for immune evasion [31].

Cancer cells of the same tumor differ in their morphology, phenotype, and functions. This intratumor heterogeneity is caused by genetic and epigenetic differences, and is also reflected in the heterogeneity of MHC-I expression. Based on IHC staining, human tumors are classified into MHC-I positive (human leukocyte antigen (HLA) class I is expressed in >75% of the cells), heterogeneous (25–75% of the cells), or negative (<25% of the cells). Garrido et al. [28] assume that these different types of MHC-I expression are linked to different stages of tumor development associated with T-cell immune selection, and have described two patterns of MHC-I expression, leukocyte infiltration, and tumor tissue architecture, which represent the early and late phases of tumor development. In the early phase (phase I, “permissive”), when cancer cells are MHC-I positive or heterogeneous in MHC-I expression, the tumors are infiltrated by T lymphocytes that are able to recognize and eliminate MHC-I positive tumor cells. The tumors can also be infiltrated by other immune cells, such as macrophages. MHC-I downregulation in tumor cells allows them to evade the attack of T cells. At the late phase (phase II, “non-permissive/encapsulated”), MHC-I negative tumor cells are encapsulated by the stroma. Immune cells do not infiltrate these tumor cells, but the stroma is infiltrated by different types of T cells and immunosuppressive cells, including Treg, macrophages, and myeloid-derived suppressor cells (MDSC).

The heterogeneity of MHC-I expression in tumors is further enhanced in metastases, where other alterations of MHC-I expression, both reversible and irreversible, can be found. Thus, metastases can have the same or different MHC-I phenotype in comparison with the primary tumor [32]. Moreover, the metastases in one patient can differ in MHC-I expression [33,34]. The frequency of MHC-I downregulation is higher in metastatic than in primary tumors [35].

## 4. PD-1/PD-L1 Blockade and Predictive Biomarkers

Many cancer patients contain potentially cytotoxic, tumor-specific T cells that are dysfunctional due to the suppression mediated by inhibitory receptors. As the interaction between the PD-1 and PD-L1 molecules contributes to this immunosuppression, its blockade can release these T cells from a state of exhaustion and enable the elimination of tumor cells. This assumption was confirmed in a mouse model after the administration of an anti-PD-1 monoclonal antibody [36].

The first clinical trial with nivolumab, a fully humanized antibody against PD-1, showed the safety of this treatment and demonstrated complete or partial responses in patients with colorectal cancer, melanoma, and RCC [37]. Several other antibodies for a PD-1/PD-L1 blockade have been developed by different companies, and this approach is being tested in numerous clinical trials as a monotherapy, or in combination with chemotherapy, radiotherapy, and other types of immunotherapy [12,38,39]. The published results of the clinical trials have evidenced durable responses in patients with both solid tumors of various origins and hematological malignancies. Remarkable objective response rates (ORR) have been found for patients with advanced treatment-refractory melanoma (32%), RCC (29%), bladder cancer (26%), and NSCLC (17%) [12,39]. The successful clinical trials led to the fast FDA approval of the PD-1/PD-L1 blockade as a second-line treatment for melanoma in 2014, NSCLC and RCC in 2015, and bladder cancer in 2016.

Despite the impressive efficacy of the PD-1/PD-L1 blockade in patients with tumors resistant to conventional cancer therapy, this anti-PD-1/PD-L1 therapy is still unsatisfactory as most patients do not respond to it. Moreover, in a study with a median duration of follow-up of 21 months, melanoma progression has been reported in approximately 25% of patients with an objective response to a PD-1 blockade [40]. In addition, this therapy can induce severe immune-related adverse events that are multiplied after combination with the CTLA-4 blockade [41]. To maximize the therapeutic benefit, biomarkers for the prediction of a response to a PD-1/PD-L1 blockade are being extensively investigated. The PD-L1 expression on tumor cells is the first possible biomarker. The clinical trials on different tumors have shown that ORR is higher in patients with PD-L1 expression than in PD-L1-negative patients, but as a portion of the latter subgroup also responded to treatment, the PD-L1 IHC alone cannot be used for the stratification of patients and in making a decision as to the eligibility for anti-PD-1/PD-L1 therapy [42].

Many human tumors are heavily infiltrated by immune cells. As cancer development is influenced by the host’s immunity, the evaluation of the number, phenotype, and spatial distribution of immune cells in tumors can provide helpful prognostic information. At present, this concept of an “immunoscore” is mostly based on the evaluation of subpopulations of T cells, particularly CD8^+^ T cells [43]. The immunoscore seems to be a superior prognostic factor in comparison to the tumor-node-metastasis (TNM) classification in early-stage colorectal cancer [44]. For cancer immunotherapy, and especially a PD-1/PD-L1 blockade where CD8^+^ T cells should be principal effector cells, such an immunoscore could also be a predictive factor because infiltrating CD8^+^ T lymphocytes are a marker of a pre-existing immunity that can be released from immunosuppression by a PD-1/PD-L1 blockade. In a study with an anti-PD-1 antibody in melanoma patients, the infiltration of CD8^+^ T cells was higher both at the invasive tumor margin and in the tumor parenchyma in the pre-treatment samples of responders compared to nonresponders [45].

Based on PD-L1 expression and the presence of tumor-infiltrating lymphocytes (TIL), human tumors have been classified into four types [46]: type I, PD-L1^+^TIL^+^ (adaptive immune resistance, where PD-L1 expression is induced by TIL); type II, PD-L1^−^TIL^−^ (immune ignorance); type III, PD-L1^+^TIL^−^ (the intrinsic induction of PD-L1 expression); and type IV, PD-L1^−^TIL^+^ (immune tolerance, where factors other than PD-L1 expression are implicated in the evasion of the host’s immunity). Tumors of type I are the most eligible for treatment with a PD-1/PD-L1 blockade, because the infiltration of TIL is evidence of a pre-existing immunity that is suppressed by PD-1/PD-L1 signaling. Unlike type 1, tumors of type IV would not probably benefit from anti-PD-1/PD-L1 therapy, as the immunosuppression is not caused by PD-L1 engagement. For tumors without TIL, both PD-L1 positive and PD-L1 negative, a PD-1/PD-L1 blockade should be combined with another treatment that induces tumor-specific T cells infiltrating tumors.

As somatic mutations generate neoantigens that can be major targets of anti-tumor T-cell immunity, mutational load has been considered as another possible predictive biomarker. Champiat, et al. [47] have shown an association of the response rate to a PD-1/PD-L1 blockade with the frequency of somatic mutations in different tumors, and have hypothesized the key role of mutational load for the success of immune checkpoint therapy. Using whole-exome sequencing, a higher nonsynonymous mutation burden has been really found in NSCLC patients who responded to an anti-PD-1 antibody in comparison to nonresponders [48]. In addition, the importance of neoantigen intratumor heterogeneity for a patient’s response has been demonstrated by a multiregion sequence analysis, which showed a greater clinical benefit in patients with a high mutational load and low neoantigen heterogeneity in comparison with a high mutational load alone [49]. For patients with colorectal cancer, the mismatch-repair status of tumors, which is associated with the frequency of somatic mutations, is an important predictive factor, as ORR after anti-PD-1 therapy was 0% in patients without a mismatch-repair deficiency and 40% in patients with this deficiency [50].

## 5. PD-1/PD-L1 Blockade and MHC-I Expression

Considering the commonly accepted key roles of CTLs in tumor eradication and that of a PD-1/PD-L1 blockade in the activation of these cells, MHC-I expression on tumor cells should be a prerequisite for successful anti-PD-1/PD-L1 therapy. However, as described above, MHC-I downregulation is frequent in human tumors. The surface expression of MHC-I molecules could thus be an important predictive biomarker. Surprisingly, special attention has not usually been devoted to MHC-I expression in studies looking for biomarkers of response to checkpoint blockade.

The expression of MHC-I genes (HLA-A, -B, and -C) in human tumors can be found in studies comparing the transcriptoms in samples taken from patients before anti-PD-1/PD-L1 therapy. In melanomas, MHC-I expression was higher (without statistical significance) in responders to therapy [51]. In another study with melanomas [52], differences in MHC-I expression have not been reported in pretreatment samples, but the expression of genes that are associated with antigen processing and presentation, including MHC-I genes, was increased in responders early during the course of anti-PD-1 therapy. Inoue et al. [53] detected the mRNA expression of one MHC-I locus—HLA-A—in melanoma patients, and showed a significantly higher expression in responders to a PD-1 blockade. They also observed that a combination of three predictive biomarkers—PD-L1, granzyme A (GZMA), and HLA-A—distinguished responders from nonresponders.

An IHC analysis of HLA-A molecules on tumor cells in melanomas revealed almost ubiquitous expression, without a statistically significant difference between responders and nonresponders [54]. However, this study also demonstrated that MHC-II (HLA-DR) expression, evaluated by IHC staining, correlated with a response to a PD-1 blockade.

An analysis of the genetic changes in melanoma samples from four patients with an acquired resistance to a PD-1 blockade suggested an association of the resistance with MHC-I downregulation [55]. After the clonal selection of resistant tumor cells, IFN-γ signaling was abrogated in two patients due to the loss of JAK1 or JAK2 kinases, and, in one patient, the gene encoding β2-microglobulin was truncated, which led to the elimination of surface MHC-I expression. Loss-of-function mutations in JAK1 or JAK2 genes can also be involved in the primary resistance to a PD-1/PD-L1 blockade. Genetic alterations in JAK1 and JAK2 have been found in different types of human tumors, with a range of 6–12% and 5–17%, respectively [56]. The abrogation of IFN-γ signaling was also associated with the primary resistance to anti-CTLA-4 therapy [57].

## 6. Utilization of PD-1/PD-L1 Blockade for Tumors with MHC-I Downregulation

The preclinical data from murine models documented that CD8^+^ T cells are crucial for the effect of a PD-1/PD-L1 blockade [58,59]. The importance of these cells was further supported by clinical trials that demonstrated the association of the benefit of anti-PD-1/PD-L1 therapy with tumor-infiltrating CD8^+^ cells [45] and a high mutational load [47,48,50]. Thus, the presentation of antigenic epitopes by MHC-I molecules on tumor cells seems to be a necessary condition for a successful PD-1/PD-L1 blockade. However, the scarce results published on this issue are ambiguous, and conclusions on the significance of MHC-I expression for anti-PD-1/PD-L1 therapy cannot be made.

Some studies suggest that antibodies against PD-1/PD-L1 could also be useful in the treatment of tumors with MHC-I downregulation. In patients with classical Hodgkin’s lymphoma (cHL), the response rates to a PD-1/PD-L1 blockade were from 65% to 87%. As the total loss of MHC-I expression on malignant cells was usually detected in about half of cHL patients, antitumor cytotoxicity was not probably caused only by CD8^+^ T lymphocytes [60]. A similar deduction is probably applicable for some other types of human tumors.

Bercovici and Trautmann have challenged the concept that the direct killing of tumor cells is a key role of T lymphocytes in an antitumor response [61]. Instead, they assume that T cells contribute to an activation of innate immune cells that eradicate the tumor. They supported their idea by experimental observation in the TC-1 tumor model, showing that after peritumoral vaccination against a tumor-specific antigen combined with the administration of IFN-α, a dynamic cooperation between CD8^+^ T cells and myeloid cells was necessary for tumor regression. Macrophages rather than CD8^+^ T cells were principal cytotoxic cells [62], despite the fact that the TC-1 cells express MHC-I molecules and are sensitive to specific immunization [63]. In another experimental study [64], combination immunotherapy induced the regression of large established tumors in three mouse transplantation models when four components were administered: an antibody specific to a surface tumor antigen, a stabilized recombinant interleukin-2, an antibody blocking the PD-1 receptor, and a powerful vaccine activating CD8^+^ T cells. This treatment stimulated strong innate and adaptive immune responses, and was also efficient against melanoma B16F10 cells with reversibly reduced MHC-I expression. For the eradication of tumors, CD8^+^ (but not CD4^+^) T cells, cross-presenting dendritic cells, macrophages, NK cells, and neutrophils were required, but the cytotoxic mechanisms were not further analyzed.

A PD-1/PD-L1 blockade can directly influence the function of other cells besides T cells, as the PD-1 receptor is expressed on B, NK, and NKT cells, and on DCs and macrophages [2]. NK cells are innate cytotoxic cells that can attack tumor cells. Their antitumor activity is regulated by signals from activating and inhibitory receptors. As MHC-I molecules interact with inhibitory receptors, tumor cells with a reduced MHC-I expression are increasingly sensitive to NK-cell cytotoxicity. NK cells with an upregulated PD-1 expression have been found in patients with Kaposi sarcoma [65] and ovarian carcinoma [66]. These cells displayed impaired degranulation and a low proliferative and cytokine response to external stimuli. Their antitumor activity was partially restored by a PD-1 blockade [66]. Thus, the PD-1 receptor could induce exhaustion in NK cells, in a way similar to T cells.

Invariant NKT (iNKT) cells are other cells with a potential for the direct killing of tumor cells. Moreover, after activation, they quickly produce large amounts of a broad range of cytokines and chemokines that stimulate strong innate and adaptive immune responses. PD-L1 expression was increased in iNKT cells from NSCLC patients [67]. The stimulation of iNKT cells from healthy donors by α-galactosylceramide (α-GalCer)-pulsed antigen-presenting cells upregulated surface PD-1 expression. A PD-1/PD-L1 blockade at the time of iNKT stimulation enhanced the T helper type 1 (Th1) cytokine production and direct cytotoxicity of iNKT cells, prevented the induction of iNKT anergy, and supported NK cell-mediated cytotoxicity [67,68,69,70]. It also reduced lung metastasis in the B16 melanoma model [68,69].

Various subpopulations of CD4^+^ T lymphocytes have been identified that mostly orchestrate the function of other immune cells, and that either augment or reduce antitumor immunity. However, some CD4^+^ T cells have been classified as effector cells capable of the direct or indirect killing of tumor cells [71,72]. In mouse tumor models, such CD4^+^ T cells were more efficient in reducing tumor growth than CD8^+^ T cells, even when the tumor cells were MHC-I positive and MHC-II negative. For this effect, cooperation with NK cells was necessary [73]. Tumor cells with MHC-II expression can be eliminated by directly cytotoxic CD4^+^ T cells. For human melanoma cells, the cytolytic activity of CD4^+^ T cells was dependent on perforin and granzyme B expression, and was only efficient against cells with high MHC-II expression. This cytotoxic effect of CT4^+^ T lymphocytes was enhanced by the blockade of PD-1 molecules [74].

Macrophages can express both PD-1 and PD-L1 molecules, but little is known about the relationship of this expression to the antitumor effect of a PD-1/PD-L1 blockade. In some tumors, PD-L1-expressing myeloid cells (including macrophages) were more abundant than tumor cells expressing PD-L1 [75,76,77], and PD-L1 expression on myeloid cells rather than tumor cells has been suggested to affect the response to a PD-1 blockade [76]. The importance of PD-L1 expression on myeloid-infiltrating cells has recently been supported by the removal of PD-L1 production from tumor cells in two mouse models [78]. In macrophages from patients chronically infected with the hepatitis C virus, the suppressed production of IL-12 was increased by a PD-1/PD-L1 blockade, which was associated with STAT-1 activation by phosphorylation [79]. A similar effect could be achieved in M2-polarized tumor macrophages that are characterized by aberrant IL-12 production, as PD-1 has been reported to suppress M1 polarization by the inhibition of STAT-1 phosphorylation, and also support M2 polarization by the augmentation of STAT-6 phosphorylation [80].

For the treatment of tumors with reversibly downregulated MHC-I expression, stimulation with IFN-γ produced by activated tumor-infiltrating immune cells or delivered as a therapeutic drug was expected to improve cancer therapy. Besides the upregulation of MHC-I molecules, the possible antitumor function of this cytokine stems from its support of a Th1 immune response, its antiangiogenic properties, and its antiproliferative and proapoptotic effects against tumor cells. However, the results of clinical trials with recombinant IFN-γ were ambiguous, and the protumorigenic effects of IFN-γ have been repeatedly demonstrated in mouse models [81,82]. As PD-L1 is also inducible on tumor cells by IFN-γ, its role in the protumoral activity of IFN-γ has been suggested, and combined therapy with IFN-γ and antibodies against PD-1/PD-L1 has been advised [82]. The benefit of combined treatment with interferons and a PD-1/PD-L1 blockade has already been verified in experimental models for IFN-α [83] and IFN-β [84].

## 7. Future Perspectives

**Figure 1:**
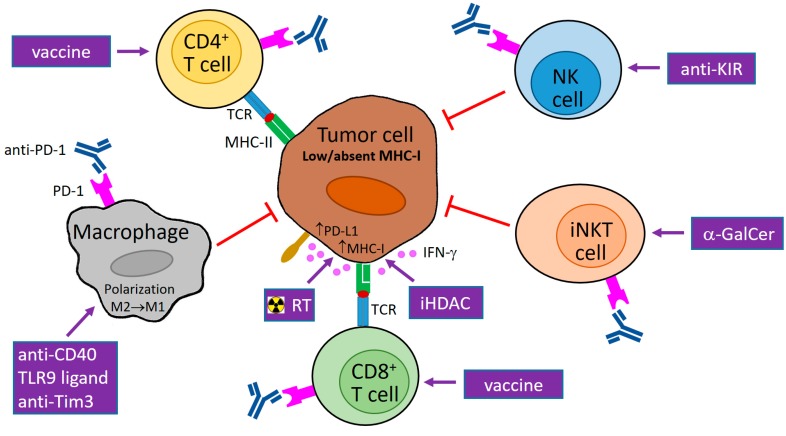
Potential usage of programmed cell death protein 1 (PD-1) blockade in combination therapy of tumors with downregulated major histocompatibility class I (MHC-I) expression. MHC-I expression in tumor cells can be reduced reversibly by epigenetic regulations or irreversibly by genetic aberrations. Abrogated interferon (IFN)-γ signaling can contribute to low MHC-I expression. The blockade of PD-1 molecules on natural killer (NK), invariant NKT (iNKT), and cluster of differentiation 4^+^ (CD4^+^) T cells supports the direct cytotoxicity of these cells to tumor cells that is not restricted by MHC-I molecules (and is dependent on MHC-II expression in the case of CD4^+^ T cells). In tumor cells with reversible MHC-I downregulation and functional IFN-γ signaling, MHC-I molecules are upregulated by IFN-γ produced by activated immune cells, e.g., iNKT cells, NK cells, and T lymphocytes stimulated by α-galactosylceramide (α-GalCer), antibody against a killer-cell immunoglobulin-like receptor (KIR), and a vaccine, respectively. As this enhancement of MHC-I expression is accompanied by PD-1 ligand 1 (PD-L1) upregulation, a PD-1/PD-L1 blockade is needed for the restoration of anti-tumor activity of cytotoxic CD8^+^ T cells, which can be augmented by tumor-specific vaccination. MHC-I expression in tumor cells is further inducible by radiotherapy (RT) and inhibitors of histone deacetylases (iHDAC). Anti-PD-1 therapy also stimulates the secretion of T helper type 1 (Th1) cytokines (including IFN-γ) and chemokines by immune cells, and could induce the polarization of tumor macrophages to the M1 phenotype, which is associated with the killing of tumor cells. This polarization can further be supported by different stimuli, e.g., CD40 agonists, toll-like receptor 9 (TLR9) ligands, and a T-cell immunoglobulin and mucin domain 3 (Tim3) blockade. TCR: T-cell receptor; M1/M2: macrophage subtype 1/subtype 2; purple arrow: activation/stimulation; red T-bar arrow: inhibition/cytotoxicity.

To improve the clinical efficacy of an antitumor PD-1/PD-L1 blockade, combinations with other therapeutic drugs or approaches are necessary. Some combined treatments are already under way [85], including combinations with other immunotherapies, e.g., vaccines or antibodies against other checkpoint receptors, and co-stimulatory molecules. As the list of potentially usable immunotherapies, either already approved for practical use or clinically tested, is extensive [86], standardized and optimized immunomonitoring will be crucial for tailoring cancer immunotherapy. With respect to MHC-I downregulation, multicolor IHC detection should particularly analyze MHC-I and PD-L1 expression on tumor cells and also on myeloid cells in the case of PD-L1, and evaluate the infiltration of tumors not only by T lymphocytes but also by myeloid cells, especially macrophages. As these cells can potentially eliminate tumor cells irrespective of MHC-I expression and predominate in some tumors, their repolarization from M2 to M1 phenotype [87], accompanied by a checkpoint blockade of PD-1/PD-L1 and/or T-cell immunoglobulin and mucin domain 3 (Tim3) molecules, might be a universal therapy for tumors heavily infiltrated by macrophages. For the treatment of tumors with reversibly downregulated MHC-I expression, a combination of a PD-1/PD-L1 blockade with the epigenetic alteration of gene expression could be beneficial. Inhibitors of histone deacetylases (HDAC) enhanced MHC-I molecules on tumor cells, even when these cells did not respond to IFN-γ stimulation [88]. HDAC inhibitors can also support antitumor immunity by other mechanisms [89], and have augmented the antitumor effect of a PD-1/PD-L1 blockade in several experimental studies [90,91,92]. The increased production of chemokines attracting T cells into tumors [92] and the elimination of MDSCs [90] have been described as contributing mechanisms. To verify the efficacy of the aforementioned approaches for the treatment of tumors with a downregulation of MHC-I expression, appropriate animal models should be developed and utilized, e.g., tumor cells lacking β2-microglobulin production, or cells with a reversible reduction of MHC-I molecules and abolished IFN-γ signaling. In the latter model, a combination with radiotherapy could be particularly useful, as it supports MHC-I expression by the induction of IFN-β production and restores the responsiveness of resistant cells to a PD-1 blockade [93]. To conclude, the focus on MHC-I downregulation in tumor cells might substantially improve the efficacy of cancer therapies including a PD-1/PD-L1 blockade (Figure 1). 

## References

[1] Ishida Y., Agata Y., Shibahara K., Honjo T. (1992). Induced expression of PD-1, a novel member of the immunoglobulin gene superfamily, upon programmed cell death. EMBO J..

[2] Agata Y., Kawasaki A., Nishimura H., Ishida Y., Tsubata T., Yagita H., Honjo T. (1996). Expression of the PD-1 antigen on the surface of stimulated mouse T and B lymphocytes. Int. Immunol..

[3] Chikuma S., Terawaki S., Hayashi T., Nabeshima R., Yoshida T., Shibayama S., Okazaki T., Honjo T. (2009). PD-1-mediated suppression of IL-2 production induces CD8^+^ T cell anergy in vivo. J. Immunol..

[4] Ahmadzadeh M., Johnson L.A., Heemskerk B., Wunderlich J.R., Dudley M.E., White D.E., Rosenberg S.A. (2009). Tumor antigen–specific CD8 T cells infiltrating the tumor express high levels of PD-1 and are functionally impaired. Blood.

[5] Chemnitz J.M., Parry R.V., Nichols K.E., June C.H., Riley J.L. (2004). SHP-1 and SHP-2 associate with immunoreceptor tyrosine-based switch motif of programmed death 1 upon primary human T cell stimulation, but only receptor ligation prevents T cell activation. J. Immunol..

[6] Dong H., Zhu G., Tamada K., Chen L. (1999). B7-H1, a third member of the B7 family, co-stimulates T-cell proliferation and interleukin-10 secretion. Nat. Med..

[7] Latchman Y., Wood C.R., Chernova T., Chaudhary D., Borde M., Chernova I., Iwai Y., Long A.J., Brown J.A., Nunes R. (2001). PD-L2 is a second ligand for PD-1 and inhibits T cell activation. Nat. Immunol..

[8] Intlekofer A.M., Thompson C.B. (2013). At the bench: Preclinical rationale for CTLA-4 and PD-1 blockade as cancer immunotherapy. J. Leukoc. Biol..

[9] Dong H., Strome S.E., Salomao D.R., Tamura H., Hirano F., Flies D.B., Roche P.C., Lu J., Zhu G., Tamada K. (2002). Tumor-associated B7-H1 promotes T-cell apoptosis: A potential mechanism of immune evasion. Nat. Med..

[10] Wang X., Teng F., Kong L., Yu J. (2016). PD-L1 expression in human cancers and its association with clinical outcomes. OncoTargets Ther..

[11] Berry S., Taube J.M. (2015). Innate vs. adaptive: PD-L1-mediated immune resistance by melanoma. OncoImmunology.

[12] Topalian S.L., Drake C.G., Pardoll D.M. (2015). Immune checkpoint blockade: A common denominator approach to cancer therapy. Cancer Cell.

[13] Parsa A.T., Waldron J.S., Panner A., Crane C.A., Parney I.F., Barry J.J., Cachola K.E., Murray J.C., Tihan T., Jensen M.C. (2007). Loss of tumor suppressor PTEN function increases B7-H1 expression and immunoresistance in glioma. Nat. Med..

[14] Lastwika K.J., Wilson W., Li Q.K., Norris J., Xu H., Ghazarian S.R., Kitagawa H., Kawabata S., Taube J.M., Yao S. (2016). Control of PD-L1 expression by oncogenic activation of the AKT-mTOR pathway in non-small cell lung cancer. Cancer Res..

[15] Marzec M., Zhang Q., Goradia A., Raghunath P.N., Liu X., Paessler M., Wang H.Y., Wysocka M., Cheng M., Ruggeri B.A. (2008). Oncogenic kinase NPM/ALK induces through STAT3 expression of immunosuppressive protein CD274 (PD-L1, B7-H1). Proc. Natl. Acad. Sci. USA.

[16] Akbay E.A., Koyama S., Carretero J., Altabef A., Tchaicha J.H., Christensen C.L., Mikse O.R., Cherniack A.D., Beauchamp E.M., Pugh T.J. (2013). Activation of the PD-1 pathway contributes to immune escape in EGFR-driven lung tumors. Cancer Discov..

[17] Dorand R.D., Nthale J., Myers J.T., Barkauskas D.S., Avril S., Chirieleison S.M., Pareek T.K., Abbott D.W., Stearns D.S., Letterio J.J. (2016). Cdk5 disruption attenuates tumor PD-L1 expression and promotes antitumor immunity. Science.

[18] Casey S.C., Tong L., Li Y., Do R., Walz S., Fitzgerald K.N., Gouw A.M., Baylot V., Gütgemann I., Eilers M. (2016). MYC regulates the antitumor immune response through CD47 and PD-L1. Science.

[19] Green M.R., Monti S., Rodig S.J., Juszczynski P., Currie T., O’Donnell E., Chapuy B., Takeyama K., Neuberg D., Golub T.R. (2010). Integrative analysis reveals selective 9p24.1 amplification, increased PD-1 ligand expression, and further induction via JAK2 in nodular sclerosing Hodgkin lymphoma and primary mediastinal large B-cell lymphoma. Blood.

[20] Steidl C., Shah S.P., Woolcock B.W., Rui L., Kawahara M., Farinha P., Johnson N.A., Zhao Y., Telenius A., Neriah S.B. (2011). MHC class II transactivator CIITA is a recurrent gene fusion partner in lymphoid cancers. Nature.

[21] The Cancer Genome Atlas Research Network (2014). Comprehensive molecular characterization of gastric adenocarcinoma. Nature.

[22] The Cancer Genome Atlas Research Network (2017). Integrated genomic and molecular characterization of cervical cancer. Nature.

[23] Bi X., Wang H., Zhang W., Wang J., Liu W., Xia Z., Huang H., Jiang W., Zhang Y., Wang L. (2016). PD-L1 is upregulated by EBV-driven LMP1 through NF-κB pathway and correlates with poor prognosis in natural killer/T-cell lymphoma. J. Hematol. Oncol..

[24] Kataoka K., Shiraishi Y., Takeda Y., Sakata S., Matsumoto M., Nagano S., Maeda T., Nagata Y., Kitanaka A., Mizuno S. (2016). Aberrant PD-L1 expression through 3′-UTR disruption in multiple cancers. Nature.

[25] Yamazaki T., Akiba H., Iwai H., Matsuda H., Aoki M., Tanno Y., Shin T., Tsuchiya H., Pardoll D.M., Okumura K. (2002). Expression of programmed death 1 ligands by murine T cells and APC. J. Immunol..

[26] Chikuma S. (2016). Basics of PD-1 in self-tolerance, infection, and cancer immunity. Int. J. Clin. Oncol..

[27] Teng M.W.L., Galon J., Fridman W.-H., Smyth M.J. (2015). From mice to humans: Developments in cancer immunoediting. J. Clin. Investig..

[28] Garrido F., Ruiz-Cabello F., Aptsiauri N. (2017). Rejection versus escape: The tumor MHC dilemma. Cancer Immunol. Immunother..

[29] Leone P., Shin E.-C., Perosa F., Vacca A., Dammacco F., Racanelli V. (2013). MHC class I antigen processing and presenting machinery: Organization, function, and defects in tumor cells. J. Natl. Cancer Inst..

[30] Garrido F., Aptsiauri N., Doorduijn E.M., Garcia Lora A.M., van Hall T. (2016). The urgent need to recover MHC class I in cancers for effective immunotherapy. Curr. Opin. Immunol..

[31] Aust S., Felix S., Auer K., Bachmayr-Heyda A., Kenner L., Dekan S., Meier S.M., Gerner C., Grimm C., Pils D. (2017). Absence of PD-L1 on tumor cells is associated with reduced MHC I expression and PD-L1 expression increases in recurrent serous ovarian cancer. Sci. Rep..

[32] Lopez-Nevot M.A., Esteban F., Ferron A., Gutierrez J., Oliva M.R., Romero C., Huelin C., Ruiz-Cabello F., Garrido F. (1989). HLA class I gene expression on human primary tumours and autologous metastases: Demonstration of selective losses of HLA antigens on colorectal, gastric and laryngeal carcinomas. Br. J. Cancer.

[33] Méndez R., Ruiz-Cabello F., Rodríguez T., Del Campo A., Paschen A., Schadendorf D., Garrido F. (2007). Identification of different tumor escape mechanisms in several metastases from a melanoma patient undergoing immunotherapy. Cancer Immunol. Immunother..

[34] Carretero R., Romero J.M., Ruiz-Cabello F., Maleno I., Rodriguez F., Camacho F.M., Real L.M., Garrido F., Cabrera T. (2008). Analysis of HLA class I expression in progressing and regressing metastatic melanoma lesions after immunotherapy. Immunogenetics.

[35] Hicklin D.J., Marincola F.M., Ferrone S. (1999). HLA class I antigen downregulation in human cancers: T-cell immunotherapy revives an old story. Mol. Med. Today.

[36] Iwai Y., Ishida M., Tanaka Y., Okazaki T., Honjo T., Minato N. (2002). Involvement of PD-L1 on tumor cells in the escape from host immune system and tumor immunotherapy by PD-L1 blockade. Proc. Natl. Acad. Sci. USA.

[37] Brahmer J.R., Drake C.G., Wollner I., Powderly J.D., Picus J., Sharfman W.H., Stankevich E., Pons A., Salay T.M., McMiller T.L. (2010). Phase I study of single-agent anti-programmed death-1 (MDX-1106) in refractory solid tumors: Safety, clinical activity, pharmacodynamics, and immunologic correlates. J. Clin. Oncol..

[38] Jiang T., Zhou C. (2015). The past, present and future of immunotherapy against tumor. Transl. Lung Cancer Res..

[39] Ma W., Gilligan B.M., Yuan J., Li T. (2016). Current status and perspectives in translational biomarker research for PD-1/PD-L1 immune checkpoint blockade therapy. J. Hematol. Oncol..

[40] Ribas A., Hamid O., Daud A., Hodi F.S., Wolchok J.D., Kefford R., Joshua A.M., Patnaik A., Hwu W.-J. (2016). Association of pembrolizumab with tumor response and survival among patients with advanced melanoma. JAMA.

[41] Larkin J., Chiarion-Sileni V., Gonzalez R., Grob J.J., Cowey C.L., Lao C.D., Schadendorf D., Dummer R., Smylie M., Rutkowski P. (2015). Combined nivolumab and ipilimumab or monotherapy in untreated melanoma. N. Engl. J. Med..

[42] Gibney G.T., Weiner L.M., Atkins M.B. (2016). Predictive biomarkers for checkpoint inhibitor-based immunotherapy. Lancet Oncol..

[43] Ascierto P.A., Capone M., Urba W.J., Bifulco C.B., Botti G., Lugli A., Marincola F.M., Ciliberto G., Galon J., Fox B.A. (2013). The additional facet of immunoscore: Immunoprofiling as a possible predictive tool for cancer treatment. J. Transl. Med..

[44] Pagès F., Kirilovsky A., Mlecnik B., Asslaber M., Tosolini M., Bindea G., Lagorce C., Wind P., Marliot F., Bruneval P. (2009). In situ cytotoxic and memory T cells predict outcome in patients with early-stage colorectal cancer. J. Clin. Oncol..

[45] Tumeh P.C., Harview C.L., Yearley J.H., Shintaku I.P., Taylor E.J.M., Robert L., Chmielowski B., Spasic M., Henry G., Ciobanu V. (2014). PD-1 blockade induces responses by inhibiting adaptive immune resistance. Nature.

[46] Teng M.W., Ngiow S.F., Ribas A., Smyth M.J. (2015). Classifying cancers based on T-cell infiltration and PD-L1. Cancer Res..

[47] Champiat S., Ferté C., Lebel-Binay S., Eggermont A., Soria J.C. (2014). Exomics and immunogenics: Bridging mutational load and immune checkpoints efficacy. OncoImmunology.

[48] Rizvi N.A., Hellmann M.D., Snyder A., Kvistborg P., Makarov V., Havel J.J., Lee W., Yuan J., Wong P., Ho T.S. (2015). Mutational landscape determines sensitivity to PD-1 blockade in non–small cell lung cancer. Science.

[49] McGranahan N., Furness A.J.S., Rosenthal R., Ramskov S., Lyngaa R., Saini S.K., Jamal-Hanjani M., Wilson G.A., Birkbak N.J., Hiley C.T. (2016). Clonal neoantigens elicit T cell immunoreactivity and sensitivity to immune checkpoint blockade. Science.

[50] Le D.T., Uram J.N., Wang H., Bartlett B.R., Kemberling H., Eyring A.D., Skora A.D., Luber B.S., Azad N.S., Laheru D. (2015). PD-1 blockade in tumors with mismatch-repair deficiency. N. Engl. J. Med..

[51] Hugo W., Zaretsky J.M., Sun L., Song C., Moreno B.H., Hu-Lieskovan S., Berent-Maoz B., Pang J., Chmielowski B., Cherry G. (2016). Genomic and transcriptomic features of response to anti-PD-1 therapy in metastatic melanoma. Cell.

[52] Chen P.-L., Roh W., Reuben A., Cooper Z.A., Spencer C.N., Prieto P.A., Miller J.P., Bassett R.L., Gopalakrishnan V., Wani K. (2016). Analysis of immune signatures in longitudinal tumor samples yields insight into biomarkers of response and mechanisms of resistance to immune checkpoint blockade. Cancer Discov..

[53] Inoue H., Park J.-H., Kiyotani K., Zewde M., Miyashita A., Jinnin M., Kiniwa Y., Okuyama R., Tanaka R., Fujisawa Y. (2016). Intratumoral expression levels of PD-L1, GZMA, and HLA-A along with oligoclonal T cell expansion associate with response to nivolumab in metastatic melanoma. OncoImmunology.

[54] Johnson D.B., Estrada M.V., Salgado R., Sanchez V., Doxie D.B., Opalenik S.R., Vilgelm A.E., Feld E., Johnson A.S., Greenplate A.R. (2016). Melanoma-specific MHC-II expression represents a tumour-autonomous phenotype and predicts response to anti-PD-1/PD-L1 therapy. Nat. Commun..

[55] Zaretsky J.M., Garcia-Diaz A., Shin D.S., Escuin-Ordinas H., Hugo W., Hu-Lieskovan S., Torrejon D.Y., Abril-Rodriguez G., Sandoval S., Barthly L. (2016). Mutations associated with acquired resistance to PD-1 blockade in melanoma. N. Engl. J. Med..

[56] Shin D.S., Zaretsky J.M., Escuin-Ordinas H., Garcia-Diaz A., Hu-Lieskovan S., Kalbasi A., Grasso C.S., Hugo W., Sandoval S., Torrejon D.Y. (2017). Primary resistance to PD-1 blockade mediated by JAK1/2 mutations. Cancer Discov..

[57] Gao J., Shi L.Z., Zhao H., Chen J., Xiong L., He Q., Chen T., Roszik J., Bernatchez C., Woodman S.E. (2016). Loss of IFN-γ pathway genes in tumor cells as a mechanism of resistance to anti-CTLA-4 therapy. Cell.

[58] Hirano F., Kaneko K., Tamura H., Dong H., Wang S., Ichikawa M., Rietz C., Flies D.B., Lau J.S., Zhu G. (2005). Blockade of B7-H1 and PD-1 by monoclonal antibodies potentiates cancer therapeutic immunity. Cancer Res..

[59] Moreno B.H., Zaretsky J.M., Garcia-Diaz A., Tsoi J., Parisi G., Robert L., Meeth K., Ndoye A., Bosenberg M., Weeraratna A.T. (2016). Response to programmed cell death-1 blockade in a murine melanoma syngeneic model requires costimulation, CD4, and CD8 T cells. Cancer Immunol. Res..

[60] Roemer M.G.M., Advani R.H., Redd R.A., Pinkus G.S., Natkunam Y., Ligon A.H., Connelly C.F., Pak C.J., Carey C.D., Daadi S.E. (2016). Classical Hodgkin lymphoma with reduced B2M/MHC class I expression is associated with Inferior outcome independent of 9p24.1 status. Cancer Immunol. Res..

[61] Bercovici N., Trautmann A. (2012). Revisiting the role of T cells in tumor regression. OncoImmunology.

[62] Thoreau M., Penny H.L., Tan K., Regnier F., Weiss J.M., Lee B., Johannes L., Dransart E., Bon A.L., Abastado J.-P. (2015). Vaccine-induced tumor regression requires a dynamic cooperation between T cells and myeloid cells at the tumor site. Oncotarget.

[63] Lin K.-Y., Guarnieri F.G., Staveley-O’Carroll K.F., Levitsky H.I., August J.T., Pardoll D.M., Wu T.-C. (1996). Treatment of established tumors with a novel vaccine that enhances major histocompatibility class II presentation of tumor antigen. Cancer Res..

[64] Moynihan K.D., Opel C.F., Szeto G.L., Tzeng A., Zhu E.F., Engreitz J.M., Williams R.T., Rakhra K., Zhang M.H., Rothschilds A.M. (2016). Eradication of large established tumors in mice by combination immunotherapy that engages innate and adaptive immune responses. Nat. Med..

[65] Beldi-Ferchiou A., Lambert M., Dogniaux S., Vely F., Vivier E., Olive D., Dupuy S., Levasseur F., Zucman D., Lebbe C. (2016). PD-1 mediates functional exhaustion of activated NK cells in patients with Kaposi sarcoma. Oncotarget.

[66] Pesce S., Greppi M., Tabellini G., Rampinelli F., Parolini S., Olive D., Moretta L., Moretta A., Marcenaro E. (2017). Identification of a subset of human natural killer cells expressing high levels of programmed death 1: A phenotypic and functional characterization. J. Allergy Clin. Immunol..

[67] Kamata T., Suzuki A., Mise N., Ihara F., Takami M., Makita Y., Horinaka A., Harada K., Kunii N., Yoshida S. (2016). Blockade of programmed death-1/programmed death ligand pathway enhances the antitumor immunity of human invariant natural killer T cells. Cancer Immunol. Immunother..

[68] Chang W.-S., Kim J.-Y., Kim Y.-J., Kim Y.-S., Lee J.-M., Azuma M., Yagita H., Kang C.-Y. (2008). Cutting edge: Programmed death-1/programmed death ligand 1 interaction regulates the induction and maintenance of invariant NKT cell anergy. J. Immunol..

[69] Parekh V.V., Lalani S., Kim S., Halder R., Azuma M., Yagita H., Kumar V., Wu L., Kaer L.V. (2009). PD-1/PD-L blockade prevents anergy induction and enhances the anti-tumor activities of glycolipid-activated invariant NKT cells. J. Immunol..

[70] Durgan K., Ali M., Warner P., Latchman Y.E. (2011). Targeting NKT cells and PD-L1 pathway results in augmented anti-tumor responses in a melanoma model. Cancer Immunol. Immunother..

[71] Van de Berg P.J., van Leeuwen E.M., ten Berge I.J., van Lier R. (2008). Cytotoxic human CD4^+^ T cells. Curr. Opin. Immunol..

[72] Quezada S.A., Peggs K.S. (2011). Tumor-reactive CD4^+^ cells: Plasticity beyond helper and regulatory activities. Immunotherapy.

[73] Perez-Diez A., Joncker N.T., Choi K., Chan W.F.N., Anderson C.C., Lantz O., Matzinger P. (2007). CD4 cells can be more efficient at tumor rejection than CD8 cells. Blood.

[74] Yan H., Hou X., Li T., Zhao L., Yuan X., Fu H., Zhu R. (2016). CD4^+^ T cell-mediated cytotoxicity eliminates primary tumor cells in metastatic melanoma through high MHC class II expression and can be enhanced by inhibitory receptor blockade. Tumor Biol..

[75] Taube J.M., Anders R.A., Young G.D., Xu H., Sharma R., McMiller T.L., Chen S., Klein A.P., Pardoll D.M., Topalian S.L. (2012). Colocalization of inflammatory response with B7-H1 expression in human melanocytic lesions supports an adaptive resistance mechanism of immune escape. Sci. Transl. Med..

[76] Herbst R.S., Soria J.-C., Kowanetz M., Fine G.D., Hamid O., Gordon M.S., Sosman J.A., McDermott D.F., Powderly J.D., Gettinger S.N. (2014). Predictive correlates of response to the anti-PD-L1 antibody MPDL3280A in cancer patients. Nature.

[77] Llosa N.J., Cruise M., Tam A., Wicks E.C., Hechenbleikner E.M., Taube J.M., Blosser R.L., Fan H., Wang H., Luber B.S. (2015). The vigorous immune microenvironment of microsatellite instable colon cancer is balanced by multiple counter-inhibitory checkpoints. Cancer Discov..

[78] Kleinovink J.W., Marijt K.A., Schoonderwoerd M.J.A., van Hall T., Ossendorp F., Fransen M.F. (2017). PD-L1 expression on malignant cells is no prerequisite for checkpoint therapy. OncoImmunology.

[79] Ma C.J., Ni L., Zhang Y., Zhang C.L., Wu X.Y., Atia A.N., Thayer P., Moorman J.P., Yao Z.Q. (2011). PD-1 negatively regulates interleukin-12 expression by limiting STAT-1 phosphorylation in monocytes/macrophages during chronic hepatitis C virus infection. Immunology.

[80] Yao A., Liu F., Chen K., Tang L., Liu L., Zhang K., Yu C., Bian G., Guo H., Zheng J. (2014). Programmed death 1 deficiency induces the polarization of macrophages/microglia to the M1 phenotype after spinal cord injury in mice. Neurotherapeutics.

[81] Zaidi M.R., Merlino G. (2011). The two faces of interferon-γ in cancer. Clin. Cancer Res..

[82] Mandai M., Hamanishi J., Abiko K., Matsumura N., Baba T., Konishi I. (2016). Dual faces of IFNγ in cancer progression: A role of PD-L1 induction in the determination of pro- and antitumor immunity. Clin. Cancer Res..

[83] Terawaki S., Chikuma S., Shibayama S., Hayashi T., Yoshida T., Okazaki T., Honjo T. (2011). IFN-α directly promotes programmed cell death-1 transcription and limits the duration of T cell-mediated immunity. J. Immunol..

[84] Kakizaki A., Fujimura T., Furudate S., Kambayashi Y., Yamauchi T., Yagita H., Aiba S. (2015). Immunomodulatory effect of peritumorally administered interferon-beta on melanoma through tumor-associated macrophages. OncoImmunology.

[85] Swart M., Verbrugge I., Beltman J.B. (2016). Combination approaches with immune-checkpoint blockade in cancer therapy. Front. Oncol..

[86] Smyth M.J., Ngiow S.F., Ribas A., Teng M.W.L. (2015). Combination cancer immunotherapies tailored to the tumour microenvironment. Nat. Rev. Clin. Oncol..

[87] Buhtoiarov I.N., Sondel P.M., Wigginton J.M., Buhtoiarova T.N., Yanke E.M., Mahvi D.A., Rakhmilevich A.L. (2011). Anti-tumour synergy of cytotoxic chemotherapy and anti-CD40 plus CpG-ODN immunotherapy through repolarization of tumour-associated macrophages. Immunology.

[88] Magner W.J., Kazim A.L., Stewart C., Romano M.A., Catalano G., Grande C., Keiser N., Santaniello F., Tomasi T.B. (2000). Activation of MHC Class I, II, and CD40 gene expression by histone deacetylase inhibitors. J. Immunol..

[89] Licciardi P.V., Karagiannis T.C. (2012). Regulation of immune responses by histone deacetylase inhibitors. Int. Sch. Res. Not..

[90] Kim K., Skora A.D., Li Z., Liu Q., Tam A.J., Blosser R.L., Diaz L.A., Papadopoulos N., Kinzler K.W., Vogelstein B. (2014). Eradication of metastatic mouse cancers resistant to immune checkpoint blockade by suppression of myeloid-derived cells. Proc. Natl. Acad. Sci. USA.

[91] Woods D.M., Sodré A.L., Villagra A., Sarnaik A., Sotomayor E.M., Weber J. (2015). HDAC inhibition upregulates PD-1 ligands in melanoma and augments immunotherapy with PD-1 blockade. Cancer Immunol. Res..

[92] Zheng H., Zhao W., Yan C., Watson C.C., Massengill M., Xie M., Massengill C., Noyes D.R., Martinez G.V., Afzal R. (2016). HDAC inhibitors enhance T-cell chemokine expression and augment response to PD-1 immunotherapy in lung adenocarcinoma. Clin. Cancer Res..

[93] Wang X., Schoenhals J.E., Li A., Valdecanas D.R., Ye H., Zhang F., Tang C., Tang M., Liu C.-G., Liu X. (2017). Suppression of type I IFN signaling in tumors mediates resistance to anti-PD-1 treatment that can be overcome by radiotherapy. Cancer Res..

